# Role of RGC-32 in multiple sclerosis and neuroinflammation – few answers and many questions

**DOI:** 10.3389/fimmu.2022.979414

**Published:** 2022-09-12

**Authors:** Alexandru Tatomir, Jacob Cuevas, Tudor C. Badea, Dafin F. Muresanu, Violeta Rus, Horea Rus

**Affiliations:** ^1^ Department of Neurology, University of Maryland, School of Medicine, Baltimore, MD, United States; ^2^ Department of Neurosciences, “Iuliu Hatieganu” University of Medicine and Pharmacy, Cluj-Napoca, Romania; ^3^ Research and Development Institute, Faculty of Medicine, Transylvania University of Brasov, Brasov, Romania; ^4^ Department of Medicine, Division of Rheumatology and Clinical Immunology, University of Maryland, School of Medicine, Baltimore, MD, United States; ^5^ Neurology Service, Baltimore Veterans Administration Medical Center, Baltimore, MD, United States

**Keywords:** RGC-32, multiple sclerosis, EAE (experimental autoimmune encephalomyelitis), radial glia, neuroinflammation, astrocyte, Th17

## Abstract

Recent advances in understanding the pathogenesis of multiple sclerosis (MS) have brought into the spotlight the major role played by reactive astrocytes in this condition. Response Gene to Complement (RGC)-32 is a gene induced by complement activation, growth factors, and cytokines, notably transforming growth factor β, that is involved in the modulation of processes such as angiogenesis, fibrosis, cell migration, and cell differentiation. Studies have uncovered the crucial role that RGC-32 plays in promoting the differentiation of Th17 cells, a subtype of CD4^+^ T lymphocytes with an important role in MS and its murine model, experimental autoimmune encephalomyelitis. The latest data have also shown that RGC-32 is involved in regulating major transcriptomic changes in astrocytes and in favoring the synthesis and secretion of extracellular matrix components, growth factors, axonal growth molecules, and pro-astrogliogenic molecules. These results suggest that RGC-32 plays a major role in driving reactive astrocytosis and the generation of astrocytes from radial glia precursors. In this review, we summarize recent advances in understanding how RGC-32 regulates the behavior of Th17 cells and astrocytes in neuroinflammation, providing insight into its role as a potential new biomarker and therapeutic target.

## Introduction

Recent years have brought an appreciable increase in our understanding of the pathogenesis of multiple sclerosis (MS), an autoimmune, demyelinating disorder of the central nervous system (CNS) with a potentially huge socioeconomic impact (1).

MS pathogenesis results from the combined action of multiple effectors, including autoreactive myelin-specific T and B cells, pro-inflammatory cytokines, macrophages, microglia, astrocytes, and the complement system (2–4). A central role is played by CD4^+^ T cells, which are thought to be primed in the periphery against myelin-specific antigens and then to migrate into the CNS, where they launch an inflammatory cascade against myelin and oligondedrocytes (OLG), leading to demyelination and eventually, in the chronic progressive phases, to axonal loss and neurodegeneration (5).

Astrocytes play vital roles in regulating physiological processes necessary for maintaining CNS homeostasis, such as synaptogenesis, neurotransmitter clearance, ion and water balance, formation and maintenance of the blood-brain barrier (BBB) and regulation of blood flow (6, 7). Astrocytes are also critical players in the pathogenesis of MS and its murine model, experimental autoimmune encephalomyelitis (EAE) by sustaining key pathological processes involved in disease initiation and progression (8–10).

First isolated from rat OLG stimulated by sublytic complement activation, RGC-32 was found to be induced by a number of growth factors, hormones, and cytokines, such as transforming growth factor (TGF)-β (11–13). RGC-32 modulates a number of cellular processes, including cell cycle regulation, cell migration, cellular differentiation, and fibrosis, and influences pathological processes such as carcinogenesis, metabolic disorders, atherosclerosis, and autoimmunity (13–15). Our work has demonstrated that RGC-32 plays an important role in the pathogenesis of EAE by regulating the differentiation of Th17 cells (16) as well as the ability of astrocytes to undergo reactive changes (17–19).

In this mini-review, we seek to summarize the most recent advances in understanding the contribution of RGC-32 to multiple sclerosis and neuroinflammation, as well as its ability to regulate astrocyte and Th17 cell biology.

## Th17 cells and their role in MS

Th17 cells differentiate from naïve CD4^+^ T cells in the presence of IL-6 and TGF-β (20). They have high pathogenic potential in light of their ability to generate pro-inflammatory cytokines, including IL-17, IL-21, IL-22 and granulocyte macrophage colony-stimulating factor (GM-CSF) (20, 21). IL-17 is particularly effective in promoting BBB disruption and in recruiting immune cells into the CNS (22, 23), while GM-CSF is highly pro-inflammatory and augments the recruitment of peripheral immune cells into the CNS (23, 24).

### RGC-32 as a key regulator of Th17 cell differentiation

Using an RGC-32 knock-out (KO) mouse model, we have been able to demonstrate that RGC-32 promotes the differentiation of Th17 cells both *in vitro* and *in vivo*. When compared to wild-type (WT) cells, CD4^+^ cells isolated from RGC-32 KO mice express lower levels of IL-17, as well as some of the transcription factors necessary for Th17 differentiation, including retinoic acid receptor-related orphan receptor gamma t (RORγt), B cell–activating transcription factor (BATF), and interferon regulatory factor 4 (IRF4) under Th17-polarizing conditions (16). On the other hand, we have observed that the differentiation of Th1, Th2, and Tregs is not affected by the lack of RGC-32. Further analysis has revealed a defect in SMAD2 and AKT phosphorylation in RGC-32 KO CD4^+^ cells, suggesting that RGC-32 preferentially facilitates the differentiation of Th17 cells in a TGF-β-dependent and independent manner (16).

Moreover, we have observed that RGC-32 KO mice develop a milder EAE phenotype than do their WT counterparts, with a lower clinical score at the peak of disease (day 14). Immunohistochemical analysis has revealed a smaller inflammatory infiltrate and fewer demyelination foci in the spinal cords of RGC-32 KO mice, and a lower number of IL-17^+^ and GM-CSF^+^ cells (16).

Interestingly, one study has shown that overexpression of RGC-32 in peripheral blood mononuclear cells (PBMC) isolated from patients with dilated cardiomyopathy augments the number of Th17 cells (25). We have also shown that B and T cells from patients with systemic lupus erythematous exhibit higher levels of RGC-32 and that overexpression of RGC-32 in human naïve CD4^+^ T cells augments the expression of IL-17 (26). These studies provide evidence of RGC-32’s role in the differentiation of human Th17 cells.

### RGC-32 as a blood-based biomarker in MS

The first evidence for a potential role of RGC-32 in MS came from experiments showing that RGC-32 is present in MS plaques in perivascular and parenchymal areas and co-localizes with CD3^+^ and CD68^+^ cells, indicating that inflammatory cells express RGC-32 in MS brains (27). In addition, astrocytes have also been found to express RGC-32 (27).

RGC-32 is also expressed in PBMC isolated from patients with relapsing-remitting MS (RRMS). The mRNA levels of RGC-32 are significantly lower in patients with relapses than in stable patients or in patients who do not respond to glatiramer acetate (GA) (28). Furthermore, RGC-32 can potentially serve as a reliable biomarker in MS, with a 90% probability of detecting relapses and 85% probability of correctly predicting responses to GA therapy (28). Moreover, *in vitro* experiments have shown that silencing RGC-32 in PBMC leads to decreased levels of Fas ligand (FasL) and SIRT1, key regulators of apoptosis, suggesting that RGC-32 can regulate immune cell survival by influencing FasL and SIRT1 expression (27).

**Figure 1:**
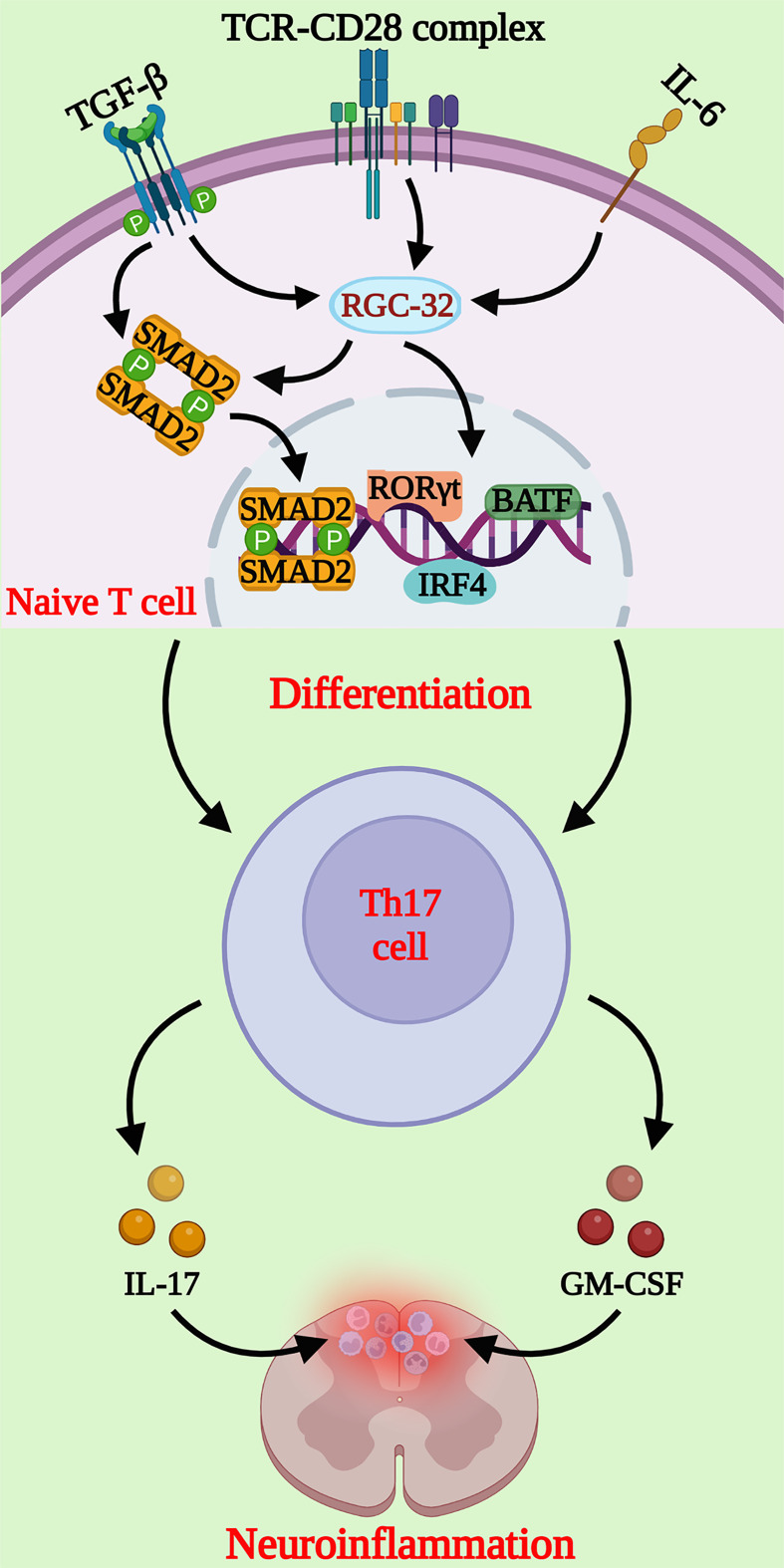
Schematic representation of the molecular pathways regulated by RGC-32 during Th17 cell differentiation. RGC-32 is upregulated in naïve CD4^+^ T cells cultured under Th17 differentiating conditions. Our studies showed that lack of RGC-32 impairs the expression of critical transcription factors involved in Th17 cell differentiation, such as the master regulator RORγt, IRF4 and BATF. The phosphorylation of SMAD2 downstream of TGF-β receptor activation might be one of the major pathways positively regulated by RGC-32 during Th17 cells generation. Th17 cells play a major role in neuroinflammatory changes at the peak of EAE mediated primarily by IL-17 and GM-CSF, that exert important chemotactic properties. (Created with BioRender.com).

Collectively, these results suggest that RGC-32 is a novel regulator of the diffrentiation of Th17 cells, making it a potential new therapeutic target in autoimmunity (**Figure 1**).

## Astrocyte emerges as an important contributor to the development and progression of MS and EAE

The morphological and molecular changes undergone by astrocytes after brain injury are collectively called reactive astrocytosis (29). Most experts in the field consider this a continuum phenomenon of transformations that can range from subtle changes in gene expression and cell metabolomics to gross morphological changes, such as cellular hypertrophy, with glial scar sitting at the extreme endpoint on this axis (30–32).

Reactive astrocytes are capable of mounting and perpetuating cellular processes leading to neuroinflammation and tissue remodeling (33). While they can exert both beneficial and detrimental effects by gain of function or loss of normal physiological properties, the net result is estimated to be pathogenic, and reactive astrocytes are currently seen as being a major contributor to MS pathogenesis (8, 34, 35).

### RGC-32 regulates the ability of astrocytes to undergo reactive changes during EAE

We have shown that the levels of glial fibrillary acidic protein (GFAP), a universal marker for reactive astrocytosis, are significantly lower in the spinal cords of RGC-32 KO mice than in WT mice under both normal conditions and in acute EAE. We have found similar results in cultured neonatal brain astrocytes, with RGC-32 KO cells having lower levels of GFAP than WT cells (18).

**Figure 2:**
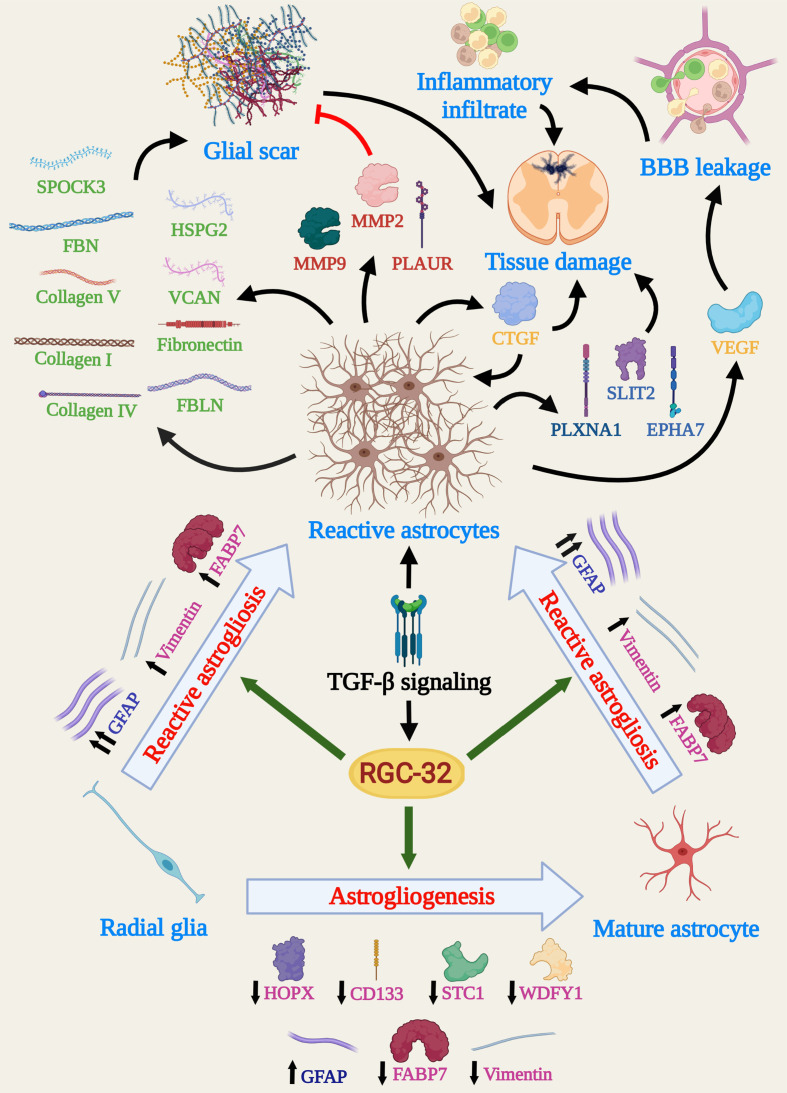
Schema depicting the main molecular and cellular processes influenced by RGC-32 in astrocytes during EAE. Data show that RGC-32 plays a major role in driving changes specific to reactive astrocytosis, such as cellular hypertrophy and glial scar formation, by favoring GFAP upregulation and synthesis of ECM components. The secretion of growth factors and AGM with BBB leakage and tissue damage potential point to a mainly pathogenic role of RGC-32, at least during acute EAE. On the other hand, RGC-32 seems to have astrogliogenic potential, since a lack of RGC-32 results in a higher number of radial glia and astrocyte precursors in adult mice. Since adult radial glial cells are a major source of reactive astrocytes in the spinal cords during EAE, it is also highly possible that RGC-32 favors this direct transition. (Created with BioRender.com).

Our team has also observed that RGC-32 KO astrocytes from the spinal cord white matter of mice with acute EAE display an elongated, bipolar morphology, reminiscent of radial glia and astrocyte progenitors, while the WT astrocytes near the inflammatory infiltrate have a reactive phenotype with body hypertrophy and process branching (17, 18). Interestingly, the number of cells that are positive for vimentin and fatty acid binding protein 7 (FABP7) and display radial glia morphology is significantly higher in RGC-32 KO mice than in WT mice on both day 0 and day 14 (18). These two markers are normally expressed by astrocyte lineage cells during brain development but can persist in mature astrocytes and adult radial glia (36–39). Their expression is increased in reactive astrocytes following brain injury (40, 41). We have observed that vimentin^+^ and FABP7^+^ radial cells have a much broader distribution in RGC-32 KO mice, whereas in WT mice they are distributed mostly around inflammatory infiltrates (18), suggesting that these cells are more likely radial glia and immature astrocytes in RGC-32 KO mice, whereas in WT mice they are vimentin- and FABP7-re-expressing reactive astrocytes. Moreover, RGC-32 KO spinal cord astrocytes display a higher proliferative index, as measured by the expression of the proliferation marker Ki-67 (18). Collectively, these results suggest that astrocytes lacking RGC-32 have an immature phenotype and display an intrinsic inability to respond to inflammation, to upregulate GFAP, and to undergo the morphological changes associated with glial scar formation at the peak of EAE (**Figure 2**).

### The immature phenotype of RGC-32 KO astrocytes translates into an impaired expression of gliotic scar components and growth factors

Glial scar plays a major role in the evolution of inflammatory lesions. While during the acute phase it might play a beneficial role by fencing the inflammatory infiltrate in and thus avoiding its spread into healthy tissue, during chronic phases it might have a rather detrimental role by inhibiting remyelination and axonal regeneration (42). However, recent advances have challenged this view and demonstrated that the glial scar can contribute to axonal regeneration (43, 44).

*In vitro* experiments have demonstrated that RGC-32 KO brain astrocytes stimulated with TGF-β produce lower levels of extracellular matrix (ECM) components such as pro-collagen I, IV, and V; fibronectin; fibrillin; and fibulin, as well as proteoglycan core proteins such as heparan sulfate proteoglycan 2 (HSPG2), versican (VCAN), and testican (SPOCK3) (17, 19). Mechanistically, RGC-32 physically associates with the transcription factor SMAD3 and is translocated to the nucleus through a process that requires SMAD3 phosphorylation and RhoA-Rho kinase activation (17). Moreover, RGC-32 KO astrocytes synthesize higher levels of enzymes involved in ECM remodeling, for example, matrix metalloproteinases (MMPs) 2 and 9, the plasminogen activator urokinase receptor (PLAUR), and tissue inhibitor of metalloproteinases 1 (TIMP1) (18). These findings point to an important role for RGC-32 in glial scar formation and remodeling.

Reactive astrocytes secrete a multitude of growth factors, with some of them having neurotrophic, reparatory effects, and others being pro-inflammatory, leading to tissue destruction (10, 45). RGC-32 KO neonatal brain astrocytes stimulated with TGF-β synthesize and/or secrete lower levels of growth factors such as connective tissue growth factor (CTGF), insulin-like growth factor 1 (IGF1), IGF binding proteins (IGFBP) 2, 3, and 6, vascular endothelial growth factor A (VEGF-A) and platelet-derived growth factor AA (PDGF-AA), than do their WT counterparts (18).

Among these proteins, VEGF and CTGF deserve special attention. VEGF plays a particular role in MS pathogenesis by facilitating BBB leakage, vascular remodeling, and immune cell trafficking (46). CTGF has been found to participate in astrocyte differentiation and activation, being able to drive reactive changes in astrocytes in an autocrine manner (47, 48). We have shown that a lack of RGC-32 impairs CTGF synthesis not only in cultured astrocytes but also in spinal cords, since RGC-32 KO mice display lower levels of CTGF^+^ astrocytes during acute EAE (18). To our knowledge, these results are the first to show that RGC-32 acts upstream of CTGF, making this molecule a major component of the TGF-β-RGC-32-CTGF axis in astrocytes.

### RGC-32 profoundly alters the transcriptomic landscape of brain astrocytes

In a quest to decipher the molecular networks underlying RGC-32’s ability to regulate astrocytic maturation and reactivity, we have performed next-generation RNA sequencing on brain astrocytes purified from WT and RGC-32 KO mice, under basal conditions and after TGF-β stimulation. We found that a lack of RGC-32 has a significant impact on the transcriptomic programs normally associated with brain development, neurogenesis, cell motility, and cell projection (19). Of special note is the fact that the differential regulation of pathways ontologically related to cell motility suggests that RGC-32 may be involved in astrocyte migration, as we have already described for other cell types such as endothelial cells and vascular smooth muscle cells (49, 50).

Functional enrichment analysis has shown that many pathways impaired by lack of RGC-32 are associated with processes such as neurogenesis and nervous system development (19). Connectivity analysis has further revealed a particular network of interconnected molecules involved in axonal guidance that is differentially regulated only in WT astrocytes (19). These axonal guidance molecules (AGM) play vital roles during brain development, providing axons with cues for normal wiring (51). In adult brains, reactive astrocytes are a major pool for AGM synthesis and secretion and, thanks to their ability to inhibit axonal regeneration and to regulate the immune system, AGM are thought to play an important role in MS pathogenesis (52–54). RGC-32 KO astrocytes have lower mRNA levels of AGM family members such as ephrin receptor A type 7 (EPHA7), plexin A1 (PLXNA1), and Slit guidance ligand 2 (SLIT2) (19). On a similar note, we have found a lower number of EPHA7/GFAP-double positive cells in the spinal cords of RGC-32 KO mice during peak EAE (19). These findings suggest the idea that the differential regulation of AGM, particularly by EPHA7, may be another major pathway by which RGC-32 facilitates reactive astrogliosis during neuroinflammation.

### RGC-32 - a missing link in astrogliogenesis?

Astrocytes are derived from radial glia, cells with pluripotent properties. In the developing spinal cord, radial glia produce intermediate precursors in the ventricular zone, which then migrate toward the mantle zone, where they proliferate before differentiating into mature astrocytes, particularly the so-called fibrous astrocytes (55, 56). A number of radial glia persist during adulthood and can serve as a pool for the generation of reactive astrocytes during EAE (57).

Immunohistochemical studies using homeodomain-only protein homeobox (HOPX), CD133, stanniocalcin-1 (STC1), and WD repeat and FYVE domain-containing protein 1 (WDFY1), four markers expressed by neural stem progenitors and radial glia (58–61), have shown that spinal cords from RGC-32 KO mice display a greater number of HOPX^+^, CD133^+^, STC1^+^, and WDFY1^+^ cells than do WT mice during acute EAE, further supporting the conclusion that in the absence of RGC-32, astrocytes remain at the stage of radial glia and progenitors (19). Furthermore, our study was the first to show that spinal cord radial glia express STC1 and WDFY1 and that their number and morphology are affected during EAE in an RGC-32 dependent manner (19). Interestingly, a recent study has found that RGC-32 is necessary for the self-renewal of neural stem cells and that a lack of RGC-32 favors neurogenesis in an *in vitro* cerebral organoid model (62).

Finally, we have shown that down-regulation of RGC-32 in cultured astrocytes reduces the nuclear translocation of signal transducer and activator of transcription 3 (STAT3) (18), which plays a key role in the gliogenic switch by activating promoters of astrocytic-specific genes such as GFAP (63).

Taken together, all these results suggest that RGC-32 may play a role in conferring on neural stem progenitors an astrogliogenic fate, thus regulating the transition of radial glia toward mature and/or reactive astrocytes, at least in the spinal cord (**Figure 2**).

## Future directions

Despite the abovementioned promising results, some questions still remain concerning exactly how RGC-32 modulates neuroinflammation:

### 1) How do astrocyte heterogeneity and species differences affect RGC-32 expression and function?

Astrocyte heterogeneity is a well-analyzed topic, and differences exist not only between species but also between spinal cord and brain astrocytes in the same species (64, 65). Therefore, in order for us to have a broader picture, additional studies are needed to determine whether our *in vivo* results can be replicated in adult mouse brains. Conversely, the transcriptomic profile of RGC-32 KO brain astrocytes should be compared with that of RGC-32 KO spinal cord astrocytes.

Similarly, in order to obtain a glimpse into the differences and similarities in RGC-32 function between mouse and human astrocytes, one should perform studies using normal human brain tissue as well as tissue isolated from MS patients to analyze the expression of proteins and genes found to be the most differentially regulated by RGC-32.

### 2) What are the other cytokines with a potential impact on the regulation of RGC-32 expression?

During acute EAE, a cocktail of various cytokines and chemokines acts at the site of inflammation (66). Therefore, a major question is: what effect(s) do other cytokines have on RGC-32 expression, beyond TGF-β? One study has reported that RGC-32 transcript are reduced by pro-inflammatory cytokines in purified brain astrocytes (67). Thus, it would be interesting to find out how the net effect of pro-inflammatory and anti-inflammatory cytokines might regulate RGC-32 expression in MS and EAE. This question leads us to wonder whether RGC-32 expression might ultimately be linked to various types of reactive astrocytes, such as the recently described pro-inflammatory, neurotoxic A1 and anti-inflammatory, protective A2 phenotypes (68), and whether the end results of anti- and pro-inflammatory influences can affect RGC-32 in such a way as to skew the balance of astrocyte reactivity toward one phenotype or another.

### 3) What role does RGC-32 play in other cell types?

RGC-32’s role in other cell types beyond the astrocyte is a fully pertinent research question to pursue. One study has found that RGC-32’s expression is activated in OLG precursor cells and promotes their proliferation after spinal cord injury (69). While we have already shown that sublytic C5b-9 can affect the OLG cell cycle by activating SIRT1 (70), additional studies are necessary to clearly delineate how RGC-32 might affect OLG during neuroinflammation and whether it might play any role in remyelination.

Microglia are CNS-resident cells with an instrumental role in driving neuroinflammation (71). Evidence suggests that RGC-32 regulates macrophage differentiation and functions in various pathologies (72–74), and since macrophages share the same developmental origin as microglia (75), we assume that RGC-32 also plays an important role in these cells. In fact, RGC-32 has been shown to be expressed by cells with a microglial morphology in MS brains (27). Thus, further studies should shed light on this issue and will help to complete the cellular and molecular puzzle centered around RGC-32 in MS and related inflammatory diseases.

## Conclusions

Understanding how exactly astrocytes interact with their environment and which molecular switches are activated at any particular point in time or space after CNS injury is crucial to decipher their pathogenic potential. RGC-32 has emerged so far as a new factor regulating astrocyte biology, since it intervenes along the whole axis of reactive astrogliosis, influencing not only the transcriptomic network but also the sheer gross morphology of reactive astrocytes. Its role in modulating other cells with crucial role in neuroinflammation, such as Th17 cells, make RGC-32 a reliable target for understanding and eventually treating MS and related diseases.

## Author contributions

AT, VR, and HR designed the study. AT, JC, TB, DM, VR, and HR wrote the manuscript. All authors contributed to the article and approved the submitted version.

## Funding

This work was supported in part by a grant from Veterans Administration Merit Award (I01BX001458 to HR), by a RO1 NS42011 grant (to HR) and by PN-III-P4-PCE-2021-0333 grant, from UEFISCDI, Romania (TB).

## Acknowledgments

We thank Dr. Deborah McClellan for editing this manuscript.

## Conflict of interest

The authors declare that the research was conducted in the absence of any commercial or financial relationships that could be construed as a potential conflict of interest.

## Publisher’s note

All claims expressed in this article are solely those of the authors and do not necessarily represent those of their affiliated organizations, or those of the publisher, the editors and the reviewers. Any product that may be evaluated in this article, or claim that may be made by its manufacturer, is not guaranteed or endorsed by the publisher.

## Abbreviations

AGM, axonal guidance molecules; BBB, blood-brain barrier; CNS, central nervous system; CTGF, connective tissue growth factor; EAE, experimental autoimmune encephalomyelitis; ECM, extracellular matrix; EPHA7, ephrin receptor type 7A; GM-CSF, granulocyte macrophage colony-stimulating factor; FBN, fibrillin; FBLN, fibulin; GFAP, glial fibrillary acidic protein; HSPG2, heparan sulfate proteoglycan 2; HOPX, homeodomain-only protein homeobox; KO, knockout; MMP, matrix metalloproteinase; MS, multiple sclerosis; PLAUR, plasminogen activator, urokinase receptor; PLXNA1, plexin A1; RGC-32, response gene to complement 32; SPOCK3, Sparc/osteonectin, cwcv and kazal-like domains proteoglycan 3; STAT3, signal transducer and activator of transcription 3; STC1, stanniocalcin-1; VEGF, vascular endothelial growth factor; VCAN, versican; WDFY1, WD repeat and FYVE domain-containing protein 1; WT, wild type.

## References

[1] Dobson R Giovannoni G. Multiple sclerosis - a review. Eur J Neurol (2019) 26:27–40. doi: 10.1111/ene.13819 30300457

[2] Nicol B Salou M Laplaud D-A Wekerle H. The autoimmune concept of multiple sclerosis. Presse Med (2015) 44:e103–12. doi: 10.1016/j.lpm.2015.02.009 25813101

[3] Mallucci G Peruzzotti-Jametti L Bernstock JD Pluchino S. The role of immune cells, glia and neurons in white and gray matter pathology in multiple sclerosis. Prog Neurobiol (2015) 127-128:1–22. doi: 10.1016/j.pneurobio.2015.02.003 25802011 PMC4578232

[4] Martin R Sospedra M Rosito M Engelhardt B. Current multiple sclerosis treatments have improved our understanding of MS autoimmune pathogenesis. Eur J Immunol (2016) 46:2078–90. doi: 10.1002/eji.201646485 27467894

[5] Engelhardt B Comabella M Chan A. Multiple sclerosis: Immunopathological heterogeneity and its implications. Eur J Immunol (2022) 52:869–81. doi: 10.1002/eji.202149757 PMC9324211 35476319

[6] Sofroniew MV Vinters HV. Astrocytes: Biology and pathology. Acta Neuropathol (2010) 119:7–35. doi: 10.1007/s00401-009-0619-8 20012068 PMC2799634

[7] Verkhratsky A Nedergaard M. Physiology of astroglia. Physiol Rev (2018) 98:239–389. doi: 10.1152/physrev.00042.2016 29351512 PMC6050349

[8] Brambilla R. The contribution of astrocytes to the neuroinflammatory response in multiple sclerosis and experimental autoimmune encephalomyelitis. Acta Neuropathol (2019) 137:757–83. doi: 10.1007/s00401-019-01980-7 PMC6483860 30847559

[9] Yi W Schluter D Wang X. Astrocytes in multiple sclerosis and experimental autoimmune encephalomyelitis: Star-shaped cells illuminating the darkness of CNS autoimmunity. Brain Behav Immun (2019) 80:10–24. doi: 10.1016/j.bbi.2019.05.029 31125711

[10] Aharoni R Eilam R Arnon R. Astrocytes in multiple sclerosis-essential constituents with diverse multifaceted functions. Int J Mol Sci (2021) 22:5904. doi: 10.3390/ijms22115904 34072790 PMC8198285

[11] Badea TC Niculescu FI Soane L Shin ML Rus H. Molecular cloning and characterization of RGC-32, a novel gene induced by complement activation in oligodendrocytes. J Biol Chem (1998) 273:26977–81. doi: 10.1074/jbc.273.41.26977 9756947

[12] Vlaicu SI Cudrici C Ito T Fosbrink M Tegla CA Rus V. Role of response gene to complement 32 in diseases. Arch Immunol Ther Exp (Warsz) (2008) 56:115–22. doi: 10.1007/s00005-008-0016-3 PMC7079747 18373239

[13] Vlaicu SI Tatomir A Anselmo F Boodhoo D Chira R Rus V. RGC-32 and diseases: The first 20 years. Immunol Res (2019) 67:267–79. doi: 10.1007/s12026-019-09080-0 31250246

[14] Vlaicu SI Tatomir A Rus V Rus H. Role of C5b-9 and RGC-32 in cancer. Front Immunol (2019) 10:1054. doi: 10.3389/fimmu.2019.01054 31156630 PMC6530392

[15] Vlaicu S Tatomir A Fosbrink M Nguyen V Boodhoo D Cudrici C. RGC-32’ dual role in smooth muscle cells and atherogenesis. Clin Immunol (2022) 238:109020. doi: 10.1016/j.clim.2022.109020 35462050

[16] Rus V Nguyen V Tatomir A Lees JR Mekala AP Boodhoo D. RGC-32 promotes Th17 cell differentiation and enhances experimental autoimmune encephalomyelitis. J Immunol (2017) 198:3869–77. doi: 10.4049/jimmunol.1602158 PMC6197070 28356385

[17] Tatomir A Tegla CA Martin A Boodhoo D Nguyen V Sugarman AJ. RGC-32 regulates reactive astrocytosis and extracellular matrix deposition in experimental autoimmune encephalomyelitis. Immunol Res (2018) 66:445–61. doi: 10.1007/s12026-018-9011-x PMC6330259 30006805

[18] Tatomir A Beltrand A Nguyen V Boodhoo D Mekala A Cudrici C. RGC-32 regulates generation of reactive astrocytes in experimental autoimmune encephalomyelitis. Front Immunol (2021) 11:608294. doi: 10.3389/fimmu.2020.608294 33569054 PMC7868332

[19] Tatomir A Beltrand A Nguyen V Courneya J-P Boodhoo D Cudrici C. RGC-32 acts as a hub to regulate the transcriptomic changes associated with astrocyte development and reactive astrocytosis. Front Immunol (2021) 12:705308. doi: 10.3389/fimmu.2021.705308 34394104 PMC8358671

[20] Patel DD Kuchroo VK. Th17 cell pathway in human immunity: Lessons from genetics and therapeutic interventions. Immunity (2015) 43:1040–51. doi: 10.1016/j.immuni.2015.12.003 26682981

[21] Bettelli E Korn T Oukka M Kuchroo VK. Induction and effector functions of T(H)17 cells. Nature (2008) 453:1051–7. doi: 10.1038/nature07036 PMC6280661 18563156

[22] Kuwabara T Ishikawa F Kondo M Kakiuchi T. The role of IL-17 and related cytokines in inflammatory autoimmune diseases. Mediators Inflammation (2017) 2017:3908061. doi: 10.1155/2017/3908061 PMC5337858 28316374

[23] Yasuda K Takeuchi Y Hirota K. The pathogenicity of Th17 cells in autoimmune diseases. Semin Immunopathol (2019) 41:283–97. doi: 10.1007/s00281-019-00733-8 30891627

[24] Pierson ER Goverman JM. GM-CSF is not essential for experimental autoimmune encephalomyelitis but promotes brain-targeted disease. JCI Insight (2017) 2:e92362. doi: 10.1172/jci.insight.92362 28405624 PMC5374070

[25] Li B Zhou W Tang X Wang W Pan J Tan M. Response gene to complement-32 promotes the imbalance of Treg/Th17 in patients with dilated cardiomyopathy. Cell Physiol Biochem (2017) 43:1515–25. doi: 10.1159/000481975 29035886

[26] Talpos-Caia A Tatomir A Nguyen V Cudrici CD Rednic S Rus HG. Response gene to complement-32 expression is upregulated in lupus T cells and promotes IL-17A expression. Lupus (2018) 5(Suppl 2):A1–A81. doi: 10.1136/lupus-2018-lsm.17

[27] Tegla CA Cudrici CD Azimzadeh P Singh AK R3 T Khan A. Dual role of response gene to complement-32 in multiple sclerosis. Exp Mol Pathol (2013) 94:17–28. doi: 10.1016/j.yexmp.2012.09.005 23000427

[28] Kruszewski AM Rao G Tatomir A Hewes D Tegla CA Cudrici CD. RGC-32 as a potential biomarker of relapse and response to treatment with glatiramer acetate in multiple sclerosis. Exp Mol Pathol (2015) 99:498–505. doi: 10.1016/j.yexmp.2015.09.007 26407760 PMC6594183

[29] Sofroniew MV. Molecular dissection of reactive astrogliosis and glial scar formation. Trends Neurosci (2009) 32:638–47. doi: 10.1016/j.tins.2009.08.002 PMC2787735 19782411

[30] Escartin C Galea E Lakatos A O’Callaghan JP Petzold GC Serrano-Pozo A. Reactive astrocyte nomenclature, definitions, and future directions. Nat Neurosci (2021) 24:312–25. doi: 10.1038/s41593-020-00783-4 PMC8007081 33589835

[31] Escartin C Guillemaud O Carrillo-de Sauvage MA. Questions and (Some) answers on reactive astrocytes. Glia (2019) 67:2221–47. doi: 10.1002/glia.23687 31429127

[32] Sofroniew MV. Astrocyte reactivity: Subtypes, states, and functions in CNS innate immunity. Trends Immunol (2020) 41:758–70. doi: 10.1016/j.it.2020.07.004 PMC7484257 32819810

[33] Giovannoni F Quintana FJ. The role of astrocytes in CNS inflammation. Trends Immunol (2020) 41:805–19. doi: 10.1016/j.it.2020.07.007 PMC8284746 32800705

[34] Correale J Farez MF. The role of astrocytes in multiple sclerosis progression. Front Neurol (2015) 6:180. doi: 10.3389/fneur.2015.00180 26347709 PMC4539519

[35] Linnerbauer M Wheeler MA Quintana FJ. Astrocyte crosstalk in CNS inflammation. Neuron (2020) 108:608–22. doi: 10.1016/j.neuron.2020.08.012 PMC7704785 32898475

[36] Sundholm-Peters NL Yang HKC Goings GE Walker AS Szele FG. Radial glia-like cells at the base of the lateral ventricles in adult mice. J Neurocytol (2004) 33:153–64. doi: 10.1023/B:NEUR.0000029654.70632.3a 15173638

[37] Barry D McDermott K. Differentiation of radial glia from radial precursor cells and transformation into astrocytes in the developing rat spinal cord. Glia (2005) 50:187–97. doi: 10.1002/glia.20166 15682427

[38] Petit A Sanders AD Kennedy TE Tetzlaff W Glattfelder KJ Dalley DA. Adult spinal cord radial glia displays a unique progenitor phenotype. PloS One (2011) 6:e24538. doi: 10.1371/journal.pone.0024538 21931744 PMC3171483

[39] Ebrahimi M Yamamoto Y Sharifi K Kida H Kagawa Y Yasumoto Y. Astrocyte-expressed FABP7 regulates dendritic morphology and excitatory synaptic function of cortical neurons. Glia (2016) 64:48–62. doi: 10.1002/glia.22902 26296243

[40] Xue P Chen L Lu X Zhang J Bao G Xu G. Vimentin promotes astrocyte activation after chronic constriction injury. J Mol Neurosci (2017) 63:91–9. doi: 10.1007/s12031-017-0961-6 28791619

[41] Kamizato K Sato S Shil SK Umaru BA Kagawa Y Yamamoto Y. The role of fatty acid binding protein 7 in spinal cord astrocytes in a mouse model of experimental autoimmune encephalomyelitis. Neuroscience (2019) 409:120–9. doi: 10.1016/j.neuroscience.2019.03.050 31051217

[42] Cregg JM DePaul MA Filous AR Lang BT Tran A Silver J. Functional regeneration beyond the glial scar. Exp Neurol (2014) 253:197–207. doi: 10.1016/j.expneurol.2013.12.024 24424280 PMC3951813

[43] Adams KL Gallo V. The diversity and disparity of the glial scar. Nat Neurosci (2018) 21:9–15. doi: 10.1038/s41593-017-0033-9 29269757 PMC5937232

[44] Bradbury EJ Burnside ER. Moving beyond the glial scar for spinal cord repair. Nat Commun (2019) 10:3879. doi: 10.1038/s41467-019-11707-7 31462640 PMC6713740

[45] Bozic I Savic D Lavrnja I. Astrocyte phenotypes: Emphasis on potential markers in neuroinflammation. Histol Histopathol (2021) 36:267–90. doi: 10.14670/HH-18-284 33226087

[46] Girolamo F Coppola C Ribatti D Trojano M. Angiogenesis in multiple sclerosis and experimental autoimmune encephalomyelitis. Acta Neuropathol Commun (2014) 2:84. doi: 10.1186/s40478-014-0084-z 25047180 PMC4149233

[47] Mendes FA Coelho-Aguiar JM Kahn SA Reis AH Dubois LG Romão LF. Connective-tissue growth factor (CTGF/CCN2) induces astrogenesis and fibronectin expression of embryonic neural cells. In Vitro. PloS One (2015) 10:e0133689. doi: 10.1371/journal.pone.0133689 26241738 PMC4524627

[48] Lu M Yan XF Si Y Chen XZ. And astrocyte-mediated inflammatory response in culture conditions. Inflammation (2019) 42:1693–704. doi: 10.1007/s10753-019-01029-7 PMC6717176 31183597

[49] Fosbrink M Cudrici C Tegla CA Soloviova K Ito T Vlaicu S. Response gene to complement 32 is required for C5b-9 induced cell cycle activation in endothelial cells. Exp Mol Pathol (2009) 86:87–94. doi: 10.1016/j.yexmp.2008.12.005 19162005 PMC2699899

[50] Vlaicu SI Tatomir A Boodhoo D Ito T Fosbrink M Cudrici C. Rgc-32 is expressed in the human atherosclerotic arterial wall: Role in C5b-9-Induced cell proliferation and migration. Exp Mol Pathol (2016) 101:221–30. doi: 10.1016/j.yexmp.2016.09.004 27619159

[51] Giger RJ Hollis 2ER Tuszynski MH. Guidance molecules in axon regeneration. Cold Spring Harb Perspect Biol (2010) 2:a001867. doi: 10.1101/cshperspect.a001867 20519341 PMC2890195

[52] Bolsover S Fabes J Anderson PN. Axonal guidance molecules and the failure of axonal regeneration in the adult mammalian spinal cord. Restor Neurol Neurosci (2008) 26:117–30. 18820406

[53] Lotfi R Nasiri Kalmarzi R Rajabinejad M Hasani S Zamani F. The role of immune semaphorins in the pathogenesis of multiple sclerosis: Potential therapeutic targets. Int Immunopharmacol (2021) 95:107556. doi: 10.1016/j.intimp.2021.107556 33756227

[54] Lee WS Lee WH Bae YC Suk K. Axon guidance molecules guiding neuroinflammation. Exp Neurobiol (2019) 28:311–9. doi: 10.5607/en.2019.28.3.311 PMC6614065 31308791

[55] Molofsky AV Deneen B. Astrocyte development: A guide for the perplexed. Glia (2015) 63:1320–9. doi: 10.1002/glia.22836 25963996

[56] Akdemir ES Huang AY Deneen B. Astrocytogenesis: Where, when, and how. F1000Res (2020) 9:233. doi: 10.12688/f1000research.22405.1 PMC7122459 32269761

[57] Bannerman P Hahn A Soulika A Gallo V Pleasure D. Astrogliosis in EAE spinal cord: Derivation from radial glia, and relationships to oligodendroglia. Glia (2007) 55:57–64. doi: 10.1002/glia.20437 17009237

[58] Li Y He ZC Zhang XN Liu Q Chen C Zhu Z. Stanniocalcin-1 augments stem-like traits of glioblastoma cells through binding and activating NOTCH1. Cancer Lett (2018) 416:66–74. doi: 10.1016/j.canlet.2017.11.033 29196129

[59] Yeo IJ Park MH Son DJ Kim JY Nam KT Hyun BK. Prdx6 inhibits neurogenesis through downregulation of WDFY1-mediated Tlr4 signal. Mol Neurobiol (2019) 56:3132–44. doi: 10.1007/s12035-018-1287-2 PMC6476867 30097850

[60] Zweifel S Marcy G Lo Guidice Q Li D Heinrich C Azim K. Hopx defines heterogeneity of postnatal subventricular zone neural stem cells. Stem Cell Rep (2018) 11:770–83. doi: 10.1016/j.stemcr.2018.08.006 PMC6135899 30174314

[61] Yang C Pu S Zhu H Qin W Zhao H Guo Z. Identification and functional characterization of CD133(+)GFAP(+)CD117(+)Sca1(+) neural stem cells. Mol Cell Biochem (2022) 477:897–914. doi: 10.1007/s11010-021-04339-3 35079926

[62] Guo Z Chen M Chao Y Cai C Liu L Zhao L. RGCC balances self-renewal and neuronal differentiation of neural stem cells in the developing mammalian neocortex. EMBO Rep (2021) 22:e51781. doi: 10.15252/embr.202051781 34323349 PMC8419700

[63] Hong S Song MR. STAT3 but not STAT1 is required for astrocyte differentiation. PloS One (2014) 9:e86851. doi: 10.1371/journal.pone.0086851 24466267 PMC3900679

[64] Yoon H Walters G Paulsen AR Scarisbrick IA. Astrocyte heterogeneity across the brain and spinal cord occurs developmentally, in adulthood and in response to demyelination. PloS One (2017) 12:e0180697. doi: 10.1371/journal.pone.0180697 28700615 PMC5507262

[65] Yang Y Jackson R. Astrocyte identity: evolutionary perspectives on astrocyte functions and heterogeneity. Curr Opin Neurobiol (2019) 56:40–6. doi: 10.1016/j.conb.2018.11.006 PMC6551291 30529823

[66] Kurschus FC. T Cell mediated pathogenesis in EAE: Molecular mechanisms. BioMed J (2015) 38:183–93. doi: 10.4103/2319-4170.155590 25900928

[67] Hasel P Rose IVL Sadick JS Kim RD Liddelow SA. Neuroinflammatory astrocyte subtypes in the mouse brain. Nat Neurosci (2021) 24:1475–87. doi: 10.1038/s41593-021-00905-6 34413515

[68] Liddelow SA Barres BA. Reactive astrocytes: Production, function, and therapeutic potential. Immunity (2017) 46:957–67. doi: 10.1016/j.immuni.2017.06.006 28636962

[69] Doi T Ogata T Yamauchi J Sawada Y Tanaka S Nagao M. Chd7 collaborates with Sox2 to regulate activation of oligodendrocyte precursor cells after spinal cord injury. J Neurosci (2017) 37:10290–309. doi: 10.1523/JNEUROSCI.1109-17.2017 PMC6596621 28931573

[70] Tatomir A Rao G Boodhoo D Vlaicu SI Beltrand A Anselmo F. Histone deacetylase SIRT1 mediates C5b-9-Induced cell cycle in oligodendrocytes. Front Immunol (2020) 11:619. doi: 10.3389/fimmu.2020.00619 32328069 PMC7160252

[71] Woodburn SC Bollinger JL Wohleb ES. The semantics of microglia activation: neuroinflammation, homeostasis, and stress. J Neuroinflamm (2021) 18:258. doi: 10.1186/s12974-021-02309-6 PMC8571840 34742308

[72] Tang R Zhang G Chen SY. Response gene to complement 32 protein promotes macrophage phagocytosis *via* activation of protein kinase c pathway. J Biol Chem (2014) 289:22715–22. doi: 10.1074/jbc.M114.566653 PMC4132778 24973210

[73] Sun C Chen SY. RGC32 promotes bleomycin-induced systemic sclerosis in a murine disease model by modulating classically activated macrophage function. J Immunol (2018) 200:2777–85. doi: 10.4049/jimmunol.1701542 PMC5893351 29507108

[74] Zhao P Wang B Zhang Z Zhang W Liu Y. Response gene to complement 32 expression in macrophages augments paracrine stimulation-mediated colon cancer progression. Cell Death Dis (2019) 10:776. doi: 10.1038/s41419-019-2006-2 31601783 PMC6786990

[75] Saijo K Glass CK. Microglial cell origin and phenotypes in health and disease. Nat Rev Immunol (2011) 11:775–87. doi: 10.1038/nri3086 22025055

